# Ubiquitin Regulation: The Histone Modifying Enzyme′s Story

**DOI:** 10.3390/cells7090118

**Published:** 2018-08-27

**Authors:** Jianlin Wang, Zhaoping Qiu, Yadi Wu

**Affiliations:** 1 Department of Pharmacology & Nutritional Sciences, University of Kentucky School of Medicine, KY 40506, USA; jianlin.wang@uky.edu (J.W.); zhaoping.qiu@uky.edu (Z.Q.); 2 Markey Cancer Center, University of Kentucky School of Medicine, Lexington, KY 40506, USA

**Keywords:** ubiquitin, epigenetic, histone modifying enzyme, protein degradation

## Abstract

Histone post-translational modifications influence many fundamental cellular events by regulating chromatin structure and gene transcriptional activity. These modifications are highly dynamic and tightly controlled, with many enzymes devoted to the addition and removal of these modifications. Interestingly, these modifying enzymes are themselves fine-tuned and precisely regulated at the level of protein turnover by ubiquitin-proteasomal processing. Here, we focus on recent progress centered on the mechanisms regulating ubiquitination of histone modifying enzymes, including ubiquitin proteasomal degradation and the reverse process of deubiquitination. We will also discuss the potential pathophysiological significance of these processes.

## 1. Introduction

Genomic DNA is tightly packaged in chromatin by both histone and non-histone proteins in the nucleus of eukaryotic cells [1]. The basic chromatin subunits, nucleosomes, are formed by wrapping 146 base pairs of DNA around an octamer core of four histones: H2A, H2B, H3, and H4 [2,3]. Whereas the nucleosomal core is compact, eight flexible lysine-rich histone tails protrude from the nucleosome, which facilitate internucleosomal contacts and provide binding sites for non-histone proteins [4]. The histones with lysine-rich tails are highly modified by histone post-translational modifications (PTMs) including acetylation, methylation, phosphorylation, ubiquitination, sumoylation, adenosine diphosphate (ADP) ribosylation, proline isomerization, biotinylation, citrullination and their various combinations [5]. These modifications constitute a unique “code” to regulate histone interactions with other proteins and thereby allow for modifications, either overcoming or solidifying, the intrinsic histone barrier to transcription. Histone modifications control dynamic transitions between transcriptionally active or silent chromatin states, and regulate the transcription of genetic information encoded in DNA (the “genetic code”) [6]. Accordingly, with these modifications, the various proteins that add, recognize and remove these PTMs, termed writers, readers and erasers, respectively, have been identified and structurally characterized. While “writer” and “eraser” enzymes modify histones by catalyzing the addition and removal of histone PTMs, respectively, “reader” proteins recognize these modified histones and “translate” the PTMs by executing distinct cellular programs. Interestingly, the stability of these “writer”, “eraser” and “reader” proteins is dynamically regulated by the ubiquitination proteasome system (UPS). The UPS alters the localization of these proteins and can promote or interfere with protein interactions, providing an additional layer to dynamic transcriptional regulation. The turnover of histone modifying enzymes through the UPS is an intrinsic cellular control mechanism that restricts an association of the enzymes with transcriptional factors and rapidly removes the enzymes from chromatin to rigorously regulate chromatin architecture and transcriptional activity.

The 76-residue protein, ubiquitin, is ubiquitously expressed and highly conserved in all eukaryotes. Ubiquitin is covalently attached to an internal lysine residue of its substrates by an enzymatic cascade, that includes an ubiquitin-activating enzyme (E1), a conjugating enzyme (E2) and a ubiquitin ligase (E3) [7]. First, an E1 recruits and activates ubiquitin by formation a thiol-ester bond between a cysteine residue of E1 and the carboxyl terminus of ubiquitin [8]. The activated ubiquitin molecule is subsequently transferred to one of several E2 ubiquitin conjugating enzymes, also through a thiol-ester linkage with ubiquitin. Subsequently, E2 mediates the transfer while the E3 provides specificity by binding to the substrate and recruiting ubiquitin to the conjugation machinery through protein-protein interaction with the E2 enzyme [9]. Most organisms have only one E1, but dozens of different E2s, and more than one thousand E3s, providing effective substrate specificity. Although the E3 ubiquitin ligase is substrate-specific, one E3 ligase may control the degradation of a variety of substrate proteins [10]. In addition, a protein could be ubiquitinated by more than one E3 ubiquitin ligase [11]. Interestingly, many substrates are modified by phosphorylation, acetylation or methylation, which act as molecular recognition signals to recruit ubiquitin E3 ligase complexes [9,12].

Ubiquitination is a reversible process and ubiquitin moieties are removed from polypeptides by deubiquitinases (DUBs), a superfamily of cysteine proteases and metalloproteases that cleave ubiquitin-protein bonds [13]. DUBs may thus counteract specific processes by removing mono-ubiquitin or poly-ubiquitin moieties from various substrates like histones, proteasome substrates and other proteins. The human genome encodes approximately 100 DUBs, which are classified into six families: (1) ubiquitin C-terminal hydrolase (UCH), (2) ubiquitin-specific processing proteases (USP), (3) Jab1/Pad1/MPN domain containing metallo-enzymes (JAMM), (4) OTU domain ubiquitin-aldehyde binding proteins (OTU), (5) Machado-Joseph disease protein domain proteases (MJDs), and (6) the monocyte chemotactic protein-induced protein (MCPIP) family [14]. In addition to deubiquitylation activities, DUBs are involved in processing newly synthesized, inactive ubiquitin precursors. By degrading ubiquitin chains, DUBs generate free ubiquitin, thus, replenishing the ubiquitin pool and maintaining the ubiquitin homeostasis [15]. Therefore, these enzymes add an extra layer in the regulation of cellular functions.

**Figure 1:**
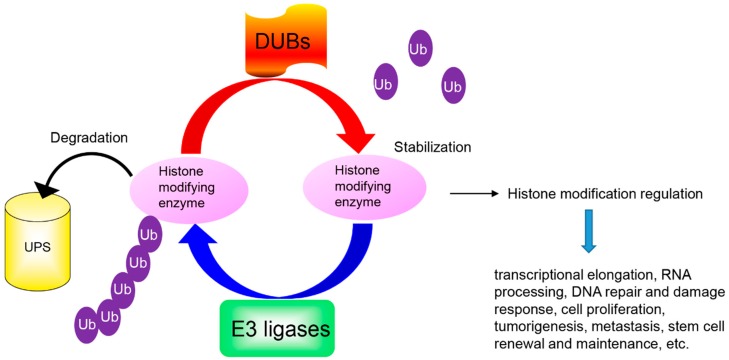
The ubiquitin proteasomal system degrades histone modifying enzymes. Histone modifying enzyme is ubiquitinated and degraded by a ubiquitin ligase (E3) while histone modifying enzyme is stabilized by deubiquitinases (DUBs) through deubiquitination. UPS = ubiquitination proteasome system; Ub = Ubiquitin.

The PTMs regulated by histone modifying enzymes play an important role in gene transcriptional activity. Rapid removal of these histone modifying enzymes from the correct histone is critical to repress or activate any target genes. The UPS controls the availability of histone modifying enzymes and indirectly alters the epigenetic code, which enables transcriptional reprogramming to control the regulation of gene expression in response to different stimuli (Figure 1). Understanding the molecular mechanism for UPS degradation of histone modifying enzymes in different pathophysiological conditions will provide new insights into how histone modifying enzymes respond to different signaling cascades and exert their diverse functions. 

## 2. Ubiquitin Proteasomal Degradation of Histone Acetylation Enzymes

Histone acetylation is a rapid and reversible process controlled by histone acetyltransferases (HATs) and histone deacetylases (HDACs). The HATs transfer acetyl groups from acetyl-coenzyme A (CoA) to the ε-amino groups of lysine residues of histone tails, which results in gene activation. HATs can be categorized into three major families, GNAT (GCN5 and PCAF), MYST (Tip60 and MOF), and p300. The HDACs remove acetyl groups from lysine residues, leading to gene silencing. Genome-wide mapping of HATs and HDACs that bind to the human genome demonstrate that these enzymes regulate the activation and repression of transcription, respectively. A dysfunctional balance between acetylation and deacetylation is clearly associated with human disease and tumorigenesis. 

### 2.1. Histone Acetyltransferases

#### 2.1.1. p300 

The p300 protein is a histone acetyltransferase and is ubiquitously expressed in the nucleus. P300 catalyzes the acetylation of lysine residues in histone proteins H2AK5, H2B (K5, K12, K15, K20), H3 (K14, K18, K23), and H4 (K5, K8, K12) [16]. In addition to histones, other nuclear proteins are also acetylated by p300, such as components of the RNA pol II complex (TFIIE and TFIIF) and a diverse group of transcription factors [17]. P300-mediated histone tail acetylation loosens up the contacts between histones and DNA, which relaxes the chromatin structure to facilitate gene transcription. P300 is essential for cell growth, proliferation, development, differentiation, cell-cycle regulation, DNA damage response, tumorigenesis, and apoptosis in many biologic processes [18,19]. 

The p300 protein level is tightly and spatially regulated through UPS degradation. P300 is degraded both in the cytoplasm and in the nucleus through distinct mechanisms. P300 turnover by UPS degradation was first identified in human cardiac myocytes [20]. Mdm2 (murine double minute 2), in the presence of active H-Ras or N-Ras, induces p300 degradation in NIH 3T3 cells [21]. Degradation of p300 is also initiated by phosphorylation of p300 at serine 1834, which is catalyzed by the cooperative action of p38 mitogen-activated protein kinases and Akt kinases [22]. The prompt degradation of p300 facilitates the sequential recruitment of downstream repair proteins for successful execution of nucleotide excision repair. Moreover, several additional E3 ligases of p300 have been identified in response to distinct upstream signals. For example, Fbx3 ubiquitin ligase promotes the degradation of p300 by the UPS in the nucleus [23]. In contrast, PML protects p300 from Fbx3-induced degradation. In addition, breast cancer metastasis suppresser 1 (BRMS1) acts as bona fide E3 ligase and as such promotes polyubiquitination and proteasome-mediated degradation of p300 [24]. Similar to the bacterial E3 IpaH family of E3 ligase, BRMS1 contains an evolutionarily conserved CXD motif that may be critical for its E3 ligase function. Mutation of this E3 ligase motif abolishes BRMS1-induced p300 polyubiquitination and degradation [24]. In agreement with this finding, inhibitory member of the apoptosis-stimulating protein of p53 (iASPP) stabilizes p300 by interfering with their BRMS1-mediated ubiquitination and enhances apoptosis upon DNA damage [25].

#### 2.1.2. PCAF (p300/CBP--Associated Factor)

PCAF interacts with CBP through its amino terminal portion and has sequence similarity with GCN5 in the carboxy-terminal half [26]. PCAF is a transcriptional co-activator with intrinsic HAT activity that acetylates free histone H3, nucleosomal H3K14 and H4K8, along with other non-histone proteins including p53 to regulate transcriptional activity [26,27]. PCAF associates with enhancer sequences to facilitate long-distance transcriptional enhancement. In addition, PCAF interacts with RNA polymerase II to maintain efficient transcriptional elongation. PCAF plays a role in multiple biological and pathogenic process such as proliferation, differentiation, apoptosis, and cell cycle progression. The E3 ubiquitin ligase Mdm2 ubiquitinates and degrades PCAF in the nucleus; devoid of Mdm2’s nuclear localization signal sequence, this enzyme is unable to degrade nuclear PCAF [28]. Interestingly, PCAF is not only a HAT, but is also a ubiquitination factor with intrinsic E3 ligase activity. PCAF could function as a ubiquitin E3 ligase for Hdm2, an oncoprotein that promotes p53 degradation, and thus play a role in regulating cellular p53 levels [29]. The potential E3 ligase activity of PCAF is within the so-called PCAF homology domain. In addition, PCAF also acts as a novel E3 ubiquitin ligase of Gli1, the final transcriptional effector of Hedgehog (Hh) signaling. PCAF, but not a mutant with a deletion of its ubiquitination domain, represses Hh signaling in response to DNA damage by promoting Gli1 ubiquitination and subsequent proteasome-dependent degradation [30]. The dual function of PCAF highlights the functional connections between cellular acetylation and ubiquitination machineries.

#### 2.1.3. HBO1 (Histone Acetyltransferase Binding to Origin Recognition Complex 1)

HBO1 belongs to the MYST family that modulates cell cycle progression, DNA replication and proliferation [31]. In general, HBO1 binds upstream of gene transcription start sites and putatively enhances gene expression. HBO1 acetylates H3K14 and histone H4 to load the origin recognition complex onto chromatin, which initiates DNA replication licensing and triggers DNA replication during the late G1 phase [32]. Ubiquitin-dependent control of the HBO1 protein contributes to cell survival during UV irradiation. HBO1 is degraded after UV-induced DNA damage to suppress cell proliferation; ATM/ATR-dependent phosphorylated HBO1 at Ser50 and Ser53 preferentially interacts with DDB2 and is ubiquitylated by CRL4 (DDB2) [33]. Interestingly, HBO1 is an unstable protein with a half-life around 3 h [32]. FBXW15 directly interacts with HBO1 to mediate its ubiquitination at K338 in the cytoplasm [32]. Phosphorylation of HBO1 mediated by mitogen-activated protein kinase 1 (Mek1) is required for FBXW15-mediated HBO1 degradation. Silencing FBXW15 blocks the Mek1-mediated HBO1 degradation [32]. Similar to PCAF, HBO1 also has intrinsic ubiquitin E3 ligase activity. HBO1 promotes destabilization of the estrogen receptor α (ERα) in breast cancers through lysine 48-linked ubiquitination [34,35]. The acetyltransferase activity of HBO1 is linked to its activity for ERα ubiquitination.

#### 2.1.4. Tip60

Tip60, a member of the MYST family, is expressed ubiquitously and is the acetyltransferase catalytic subunit of the human NuA4 complex [36]. Tip60 specifically targets H2AK5, H4K16 as well as other histone proteins. Tip60 plays an important role in many processes, such as cellular signaling, DNA damage repair, transcription and cellular cycling. Aberrant expression of Tip60 promotes or suppresses tumorigenesis in colon, breast and prostate tumors, depending on the tumor type. It is known that Tip60 is turned over in cells by the UPS. UHRF1 (Ubiquitin-like containing PHD and RING domain 1) co-localize with Tip60, and down-regulation of UHRF1 enhances Tip60 expression [37]. By contrast, Tip60 is stabilized in normal cells by UHRF2 ubiquitination and acts downstream of UHRF2 to regulate H3K9ac and H3K14ac expression [38]. Under non-stressed conditions, activating transcription factor-2 (ATF2), in cooperation with the CUL3 ubiquitin ligase promotes degradation of Tip60 [39]. Another important E3 ligase, Mdm2, interacts physically with Tip60 and induces ubiquitination and proteasome-dependent degradation [40]. Recent proteomic analyses further identified EDD1 (E3 identified by differential display), an E3 ligase generally overexpressed in cancers as a novel interacting partner of Tip60 [41]. EDD1 negatively regulates Tip60’s stability through the proteasome pathway. Interestingly, As3^+^ can bind directly to the zinc-finger motif of Tip60 in vitro and exposure to As3^+^ results in a dose-dependent decrease in Tip60 protein level via the UPS [42]. However, the mechanism used by As3^+^ to regulate Tip60 protein levels remains unknown. Recent studies revealed that ubiquitin-specific protease 7 (USP7) interacts with and deubiquitinates Tip60 both in vitro and in vivo. USP7 deubiquitinase activity is required for the stabilization of Tip60 in order to operate an effective p53-dependent apoptotic pathway in response to genotoxic stress and is central to the development and maintenance of the T regulatory (Treg) cell lineage and adipocyte differentiation [43,44,45,46]. The interaction between activating transcription factor 3 (ATF3) and Tip60 increases the Tip60 stability by promoting USP7-mediated deubiquitination of Tip60 [47]. Knockdown of ATF3 expression leads to a decreased Tip60 expression and accumulated DNA lesions and increased cell sensitivity to irradiation.

### 2.2. Histone Deacetyltransferases

#### HDAC1/HDAC2

HDAC1, a class I histone deacetyltransferase, is degraded by the UPS. HDAC1 protein levels are degraded robustly between 3 and 4 h after hormone stimulation as a result of ubiquitination. Destruction of HDAC1 is a common event in transcriptional regulation of nuclear receptors. For example, HDAC1 turnover is increased after glucocorticoids stimulation [48]. In addition, E3 ubiquitin ligase Mdm2 associates with and ubiquitinates HDAC1 at the active promoter in response to androgen [49]. Interestingly, the deacetylase activity of HDAC1 is also required to enhance Mdm2-mediated androgen receptor (AR) ubiquitination. Simultaneous degradation of HDAC1 and AR by Mdm2 confers protein destabilization and provides an additional mechanism for AR and HDAC1 regulation. Mdm2 also induces ubiquitination of HDAC1 in vascular calcification (VC). Under calcification-inducing conditions, proteasomal degradation of HDAC1 precedes VC and it is mediated by the Mdm2 E3 ubiquitin ligase that initiates HDAC1 K74 ubiquitination [50]. In addition, HDAC1 directly interacts with the carboxyl terminal region of Chfr, an E3 ubiquitin ligase, which contributes to the mitotic checkpoint. Chfr ubiquitinates HDAC1 in vitro and in vivo. Overexpression of Chfr enhances HDAC1 degradation, leading to an upregulation of p21 and the metastasis suppressors KAI1 and E-cadherin [51]. Recently, it was reported that the CUL3–REN E3 ubiquitin ligase complex also triggers HDAC1 recruitment and degradation and, consequently, Gli1 hyperacetylation, which results in inhibition of Gli1’s transcriptional activity [52]. Valproic acid (VPA), an inhibitor of Class I and II HDAC enzymes, not only inhibits HDAC catalytic activity but also triggers proteasome-mediated degradation of HDAC2 [53]. The E2 ubiquitin conjugase Ubc8 and the E3 ligase RLIM account for the degradation of HDAC2, which contributes to basal turnover of HDAC2 and is differentially regulated by VPA [53]. Interestingly, both VPA and Trichostatin A (TSA) treatment induce Ubc8 gene expression, whereas only TSA simultaneously reduces RLIM protein levels and therefore fails to induce HDAC2 degradation. Mule (Mcl-1 ubiquitin ligase E3), a HECT domain ubiquitin ligase, also specifically targets HDAC2 for ubiquitination and degradation [54]. Accumulation of HDAC2 in Mule-deficient cells leads to compromised p53 acetylation as well as crippled p53 transcriptional activation, accumulation, and apoptotic response upon DNA damage. Interestingly, cigarette smoke extract exposure leads to phosphorylation of HDAC2 by a casein kinase II (CKII)-mediated mechanism, decreased HDAC2 activity, and increased ubiquitin-proteasome-dependent HDAC2 degradation [55]. CKII and proteasome inhibitors stabilize HDAC2 from its degradation. In contrast, ubiquitin-specific peptidase 4 (USP4) interacts directly with and deubiquitinates HDAC2, leading to a stabilization of HDAC2 [56]. Another DUB, USP17, deubiquitinates and stabilizes the protein level of HDAC2. HDAC2 is excessively ubiquitinated and degraded in the proteasome because of low expression of USP17 in cigarette smoke extract-exposed airway epithelial cells and macrophages [57]. Furthermore, over-expression of USP17 attenuates the degradation of HDAC2 induced by cigarette smoke extract.

## 3. Ubiquitin Regulation of Histone Methylation Enzymes

Methylation of lysine residues on histones was first identified in the 1960s. Histone lysines can have four states of methylation and occur at different lysine sites. Histones H2B lysine 5 (H2BK5), H3K4, H3K9, H4K20, H3K27, H3K36, and H3K79 are subject to unmethylated, mono-methylation (me1), di-methylation (me2), or tri-methylation (me3) on the ε-amino groups of lysine residues. These lysine methylations change the chromatin structure to regulate gene transcription. Histone lysine methylation is a reversible modification and is maintained by the balance lysine methyltransferases (KMTs) and lysine demethylases (KDMs). The KMTs recruit S-adenosyl methionine (SAM) as a cofactor and catalyze the addition of methyl groups to lysine residues through the SET domain. The KMTs are grouped into several families: KMT1-3, KMT 5-7, KMT4/DOT1, as well as others. The KDMs include the flavin adenine dinucleotide- (FAD-) dependent monoamine oxidase family (KDM1/LSD (Lysine-Specific Demethylase)), the Jumonji C domain-containing demethylase (JMJD) families (KDM2-6), and others. Methylation of H3K4, H3K36, and H3K79 usually correlate with gene activation, whereas methylation of H3K9, H3K20, H3K27, and H3K56 are associated with transcriptional silencing.

### 3.1. Histone Methylation Enzymes

#### 3.1.1. SETD2/SETD3 (The SET-Domain Methyltransferase 2/3)

SETD2 is generally recognized as the only human gene responsible for trimethylation on lysine 36 of Histone H3 (H3K36), which recruits protein complexes that carry out a variety of processes, including transcriptional elongation, RNA processing, DNA repair and damage response, and polycomb silencing, all of which establish the impact of this histone modification [58,59]. Loss of SETD2 causes regional genomic instability, RNA processing defects, and intragenic transcription initiations. SETD2 functions as a tumor suppressor in cancer progression. In breast cancer, SETD2 expression levels are negatively associated with increasing tumor stage [58]. In gliomas and clear cell renal cell carcinoma, SETD2 is highly mutated [58]. SETD2 has a short half-life [60]. The binding between the WW domain of SETD2 and the C-terminal domain (CTD) of RNA polymerase II (RNAPII) protects SETD2 from degradation. Interestingly, removal of the SETD2-Rpb1 interacting (SRI) domain stabilizes SETD2 in addition to uncoupling SETD2 from the CTD. Thus, the SRI domain contains a degradation signal that becomes exposed when SETD2 is not CTD-bound [60]. A recent study identified SPOP, a key subunit of the CUL3 ligase complex, as a binding partner for SETD2 that mediates its turnover by the proteasome [61]. The SPOP/CUL3 complex is responsible for SETD2 polyubiquitination both in vivo and in vitro. Modulation of SPOP expression confers differential H3K36me3 on SETD2 target genes, and induces H3K36me3-coupled alternative splicing events.

SETD3 is a novel histone H3K4 and H3K36 methyltransferase with transcriptional activation activity. SETD3 levels are increased in human liver cancer cells [62]. Overexpression of SETD3 in liver cancer cells promotes cell proliferation and tumorigenesis and SETD3 protein levels correlate with high malignancy and poor prognosis in liver tumors. The SETD3 levels display a dynamic cell cycle profile. SETD3 levels are regulated in a glycogen synthase kinase-3 β (GSK-3β)- and F-Box and WD Repeat Domain Containing 7 (FBXW7) β -dependent manner in the cytoplasm. GSK-3β-mediated phosphorylation and FBXW7β-mediated ubiquitination of SETD3 are required for its proteolysis [62].

#### 3.1.2. PR-Set7/Set8 (The SET-Domain Methyltransferase PR-Set7)

PR-Set7/Set8 (also known as SET8, SETD8 or KMT5A) is a cell-cycle-regulated enzyme that monomethylates the lysine 20 of histone H4 (H4K20) [63]. PR-Set7 plays an essential role in mammalian cell cycle progression, transcriptional regulation, DNA repair, genome stability and tumor metastasis [64]. Set8 and monomethylated H4K20 are virtually undetectable during G1 and S phases of the cell cycle but increase in late S and in G2 [65,66]. A timely destruction of this enzyme during S phase is mediated by ubiquitin-mediated proteolysis and requires the interaction of the enzyme with the DNA replication factor proliferating cell nuclear antigen (PCNA) through a conserved PCNA-interacting (PIP) motif located upstream of the catalytic SET domain [67]. PCNA serves as a cofactor to promote PR-Set7 interaction with the CRL4^cdt2^ E3 ubiquitin ligase, which earmarks PR-Set7 for ubiquitylation and degradation during S phase or upon DNA damage [68,69]. PCNA-mediated degradation of mammalian PR-Set7 is essential for proper cell-cycle progression [66,70]. In addition to the CRL4^cdt2^ pathway, the anaphase-promoting complex (APC)^Cdh1^ and the F-box proteins Skp2 and β-TRCP of SCF ubiquitin E3 ligase complexes are reported to regulate PR-Set7 stability in human cells [67]. Phosphorylation of S28 in PR-Set7 by the cyclin-dependent kinase 1 (CDK1)/cyclinB complex stabilizes PR-Set7 by directly inhibiting its interaction with the APC [70]. In contrast, dephosphorylation of S29 during late mitosis by the Cdc14 phosphatases is required for APC^cdh1^-mediated ubiquitination of PR-Set7 and subsequent proteolysis. Set8 interacts with the β-TRCP E3 ligase complex including Skp1 and Rbx1 [71,72]. Depletion of either Cullin1, Cullin4 or endogenous β-TRCP extends the half-life of endogenous Set8 proteins and increases the level of the Set8 protein. CKI (casein kinase I) functions as a key upstream kinase to phosphorylate Set8 at Ser253 and subsequently triggers its destruction by β-TRCP [71]. Therefore, Set8 is governed by Skp2 and β-TRCP in the G1 phase, whereas Set8 destruction is controlled by CRL4^cdt2^ in the S phase. How these ligases fine-tune the timely destruction of Set8 to ensure proper cell cycle progression is unknown.

#### 3.1.3. EZH2 (Enhancer of Zeste Homolog 2)

**Figure 2:**
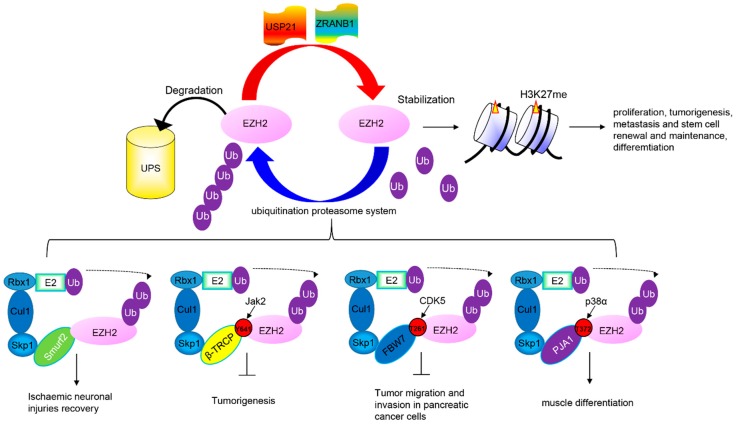
The regulation of EZH2 and related pathologies by the ubiquitin proteasomal system. Composition of the E3 complex and DUB targets EZH2. The Skp-1- Cullin-1-F-Box (SCF) E3s are multimeric E3 ligases that mediates the ubiquitin (Ub) transfers from E2 conjugating enzymes to EZH2.

EZH2 is a critical enzymatic subunit of the polycomb repressive complex 2 (PRC2), which silences gene transcription by trimethylating histone H3 (H3K27) to mediate gene repression [73]. Overexpression of EZH2 promotes cell proliferation, tumorigenesis, metastasis, and stem cell renewal and maintenance [74]. EZH2 is mutated or highly expressed in many types of cancer, including lymphoma, melanoma, prostate cancer, and breast cancer [75]. The EZH2 protein is subject to ubiquitin-dependent degradation by several E3 ligases (Figure 2). First, Smurf2 as the E3 ubiquitin ligase is responsible for the polyubiquitination and proteasome-mediated degradation of EZH2, which is required for neuron differentiation [76]. Second, EZH2 is a novel component and substrate of the SCF E3 ubiquitin ligase β-TRCP (FBXW1) [77]. β-TRCP ubiquitinates EZH2 while Janus kinases 2 (Jak2)-mediated phosphorylation on Y641 directs the β-TRCP-mediated EZH2 degradation. Silencing of β-TRCP or inhibition of Jak2 results in EZH2 stabilization with an attendant increase in H3K27 trimethylation activity. Consistent with this, endogenous EZH2(Y641) mutants exhibit increased EZH2 stability and H3K27me3 hyperactivity in lymphoma cells. Third, EZH2 is a bona fide substrate of FBXW7 in pancreatic cancer cells [78]. EZH2 phosphorylation at Thr 261 by CDK5 kinase is required for FBW7-mediated degradation. FBXW7 suppresses EZH2 activity and inhibits tumor migration and invasion via degradation of EZH2 in pancreatic cancer cells. Fourth, MYOD-induced E3 ubiquitin ligase Praja1 (PJA1) is involved in regulating EZH2 levels upon p38α activation in differentiating muscle cells [79]. The p38α kinase promotes EZH2 degradation through phosphorylation of threonine 372. Premature degradation of EZH2 in proliferating myoblasts is prevented by low levels of PJA1, its cytoplasmic localization and the lower activity with unphosphorylated EZH2. More importantly, FOXP3 accelerates EZH2 protein degradation through the polyubiquitination-proteasome pathway by enhancing the transcription of PJA1 directly [80]. Finally, EZH2 is a substrate at the COOH terminus of Hsp70-interacting protein (CHIP) [81]. CHIP triggers EZH2 degradation through ubiquitination. Recently, it was reported that the stability of EZH2 was also regulated by long noncoding RNA. Angelman syndrome chromosome region (ANCR) modulates the stability of EZH2, and hence suppresses the invasion and metastasis of breast cancer cells [82]. ANCR potentiates the CDK1-EZH2 interaction, which then increases the extent of phosphorylation at the Thr-345 and Thr-487 sites of EZH2, facilitating EZH2 ubiquitination and degradation. For the reverse process, EZH2 is stabilized by deubiquitination. Ubiquitin-specific protease (USP21) deubiquitinates EZH2 and stabilizes it. USP21 is upregulated in bladder cancer (BC) and ectopic expression of USP21 is closely associated with tumor size and metastasis [83]. USP21 facilitates cell proliferation, epithelial-mesenchymal transition and metastasis in bladder carcinoma cell lines. ZRANB1, an ovarian tumor protease (OTU) family member, also functions as an EZH2 deubiquitinase [84]. ZRANB1 binds, deubiquitinates, and stabilizes EZH2. Depletion of ZRANB1 in breast cancer cells results in EZH2 destabilization and growth inhibition (Figure 2).

### 3.2. Histone Demethylation Enzymes

#### 3.2.1. JMJD2A (Jumonji Domain 2)

JMJD2A/KDM4A is the first identified histone lysine demethylase that demethylases trimethylated residues and targets H3K9 and H3K36 [85]. The JMJD2 family consists of the three ∼130-kDa proteins (JMJDA, JMJDB and JMJDC) and JMJD2D/KDM4D, which is half the size and lacks the double PHD and Tudor domains that are epigenome readers and are present in the other KDM4 proteins [86]. JMJD2A is implicated in replication timing and genomic stability, DNA damage response, cellular differentiation, and animal development [85]. Various studies have shown that JMJD2A is overexpressed in breast, colorectal, lung, prostate, and other tumors and is required for efficient cancer cell growth [87]. The degradation of JMJD2A is regulated by different F-box-containing SCF ubiquitin ligase complexes. First, JMJD2A turnover is coordinated through the Skp1-Cul1-FBXL4 ubiquitin ligase [88]. The protein degradation of JMJD2A is also regulated by FBXW2 ubiquitin ligase [88]. In addition, FBXO22 ubiquitin ligase complex controls the activity of JMJD2A by targeting it for proteasomal turnover [89]. FBXO22 functions as a receptor for JMJD2A by recognizing its catalytic JmjN/JmjC domains via its intracellular signal transduction (FIST) domain. Modulation of FBXO22 levels by RNA interference or overexpression leads to increased or decreased levels of JMJD2A, respectively. In response to DNA damage, JMJD2A is degraded by the proteasome in an RNF8-dependent manner [90]. RNF8-dependent degradation of JMJD2A regulates DNA repair by controlling the recruitment of 53BP1 at DNA damage sites.

#### 3.2.2. LSD1 (Lysine-Specific Demethylase 1)

LSD1, the first identified histone demethylase, functions as an epigenetic regulator through an amine oxidase reaction mainly by removing H3K4 mono-/di-methylation, an activation marker of transcription [91,92]. High levels of the LSD1 have been found in leukemia, non-small cell lung, pancreatic, prostate, and breast cancers [93,94,95,96]. Overexpression of LSD1 is associated with tumor aggressiveness, metastasis, recurrence, and drug resistance and is regarded as a biomarker of poor prognosis [97,98]. LSD1 participates in different protein complexes that modulate distinct molecular targets to induce metastasis and cancer stem cells (CSC)s in a variety of cancers [99,100,101,102,103]. For example, we demonstrated that LSD1 interacts with Snail1 and promotes breast cancer metastasis through downregulation of CDH1 [104]. 

LSD1 is regulated at transcriptional and post-translational levels. We and others showed that LSD1 is under a tight control by the UPS [94,104,105,106]. We recently found that USP28 is the LSD1 deubiquitinase that stabilizes the LSD1 protein [106]. In addition, USP22 can also stabilize LSD1 through GSK-3β-mediated phosphorylation [94]. Furthermore, USP7 inhibits LSD1 ubiquitination and stabilizes LSD1in glioma [107]. USP7-LSD1 affects glioma cell proliferation and invasion. However, the bona fide E3 ligase responsible for LSD1 degradation remains largely unknown. Although Jade2 has been reported to act as an E3 ligase to destabilize LSD1 during neurogenesis [108], this observation is controversial and requires further validation, given that Jade2 is a transcription factor and that Jade2-mediated LSD1 ubiquitination is dependent on the PHD zinc finger of Jade2 rather than a classical ring finger found in almost all E3 ubiquitin ligases [108,109]. Notably, LSD1 harbors canonical (I/L)Q motifs typical for the recognition and ubiquitination by F-box protein [110]. Therefore, it is plausible that the ubiquitination of LSD1 is mediated by an F-box family E3 ligase. Identification of such an E3 ligase and the corresponding mechanism will provide new windows for therapeutic targeting of LSD1. 

## 4. Ubiquitin of Histone Arginine Methylation Enzymes

Histone arginine methylation also occurs in many arginine sites: histone H3 arginine 2 (H3R2), H3R8, H3R17, H3R26, and H4R3 undergo monomethylation (me1), symmetrical dimethylation (me2s), or asymmetrical dimethylation (me2a) on the guanidinyl groups of arginine residues. The N-arginine methyltransferases (PRMTs) are a class of enzymes that transfer a methyl group from SAM to the guanidino nitrogen of arginine. PRMTs generate three arginine methylation forms: monomethylarginine (MMA), asymmetric dimethylarginine (aDMA), and symmetric dimethylarginine (sDMA). Human PRMTs are composed of nine members that are categorized into three groups based on the type of arginine methylation reaction each member catalyzes. Type I is comprised of PRMT1, PRMT2, PRMT3, PRMT4/ coactivator-associated arginine methyltransferase 1 (CARM1), PRMT6, and PRMT8; these catalyze both mono-methyl and asymmetric dimethyl arginine reactions. The type II group is made up of two members, PRMT5 and PRMT9, which catalyze both mono-methyl arginine and symmetric dimethyl arginine. Finally, PRMT7 is, at this point, considered the only bona fide type III methyltransferase and can generate only mono-methyl arginines. Many studies demonstrated that PRMTs regulate a wide range of genetic programs and cellular processes including cell cycle, RNA splicing and differentiation. Although the consequence of lysine methylation is relatively well studied, the role of PRMT action is poorly understood.

### 4.1. PRMT1

PRMT1 is responsible for a substantial percentage of methylated arginine residues. Specifically, asymmetric dimethylation on H4R3 by PRMT1 is involved in transcriptional activation, thereby driving oncogenic pathways. PRMT1 is an important regulator of cell proliferation, progenitor maintenance, and tumor metastasis. PRMT1 is polyubiquitylated for proteasome degradation with a half-life of approximately 4 h in lung epithelial cells [111]. FBXL17 mediates PRMT1 polyubiquitination at K117. FBXL17 specifically binds PRMT1 via a unique motif IkxxxIK. The acetylation/deacetylation status of the lysine residues within the motif determines FBXL17 binding thereby triggering PRMT1 protein degradation. The tripartite motif 48 (TRIM48) has a RING-finger motif with E3 ubiquitin ligase activity, and belongs to the TRIM family. TRIM48 promotes K48-linked polyubiquitination and degradation of PRMT1 [112]. Using phage display and the orthogonal UB transfer (OUT) screen, PRMT1 was identified as a potential substrate of the U-box E3 ligase E4B and CHIP [113]. However, the detailed mechanisms of how E4B and CHIP regulate PRMT1 stability need further investigation. Interestingly, PRMT1 regulates E3 ligase activity through arginine methylation. Smurf2 is a substrate of PRMT1 [114] and methylation of Smurf2 by PRMT1 regulates Smurf2 stability and controls TGF-β signaling. Another E3 ubiquitin ligase, TNF receptor-associated factor 6 (TRAF6), is also methylated by PRMT1, and this arginine methylation inhibits TRAF6’s ubiquitin ligase activity, reducing activation of toll-like receptor signaling [115].

### 4.2. PRMT4

PRMT4, more commonly known as CARM1, is involved in the regulation of a number of cellular processes including transcription, pre-messenger RNA (mRNA) splicing, and cell cycle progression. CARM1 expression is dysregulated in colorectal, prostate and breast cancer. CARM1 methylates the chromatin-remodeling SWI/SNF core subunit, BAF155, in the arginine 1064 residue [116]. This methylation of BAF155 is associated with breast cancer recurrence and metastasis, indicating that CARM1 plays an important role in breast cancer progression through BAF155. Accordingly, CARM1-induced tumorigenic effects and its expression is increased in invasive breast cancer, and correlates with a high tumor grade [117]. Notably, CARM1 stability is regulated by the Skp2-containing SCF (Skp1-cullin1-F-box protein) E3 ubiquitin ligase in the nucleus, but not in the cytoplasm, under nutrient-rich conditions [118,119]. With nutrient deprivation, AMP-activated protein kinase (AMPK) induces phosphorylation of FOXO3a in the nucleus, which in turn transcriptionally represses Skp2. Consequently, this repression of Skp2 leads to increased levels of CARM1 protein and a subsequent increase in histone H3 Arg17 dimethylation [119]. Interestingly, high-glucose treatment increases CARM1 ubiquitination [120]. Whether high-glucose treatment increases the Skp2 activity requires further investigation. In addition, peroxide (H_2_O_2_) treatment decreases CARM1 protein stability in murine lung epithelial (MLE12) cells, which impedes cell migration through a downregulation of GSK-3β. Protein kinase GSK-3β protects CARM1 from ubiquitin proteasomal degradation by catalyzing CARM1 T132 phosphorylation [121].

## 5. Ubiquitination of Histone Modification Readers

### BRD4 (Bromodomain Containing 4)

Histone modifications are recognized by proteins containing distinct recognition domains, which act as “readers” and bind to different histone modifications [122]. For example, the bromodomain acts as a lysine acetylation “reader” of modified histones that mediate signaling transduction changes in gene regulatory networks [123]. The bromodomain and the extra-terminal domain (BET) family recognize acetylated lysine residues in histones H3 and H4 [124]. BRD4 is a member of the BET family that carries two bromodomains. BRD4 functions as a transcriptional coactivator and plays critical roles in a variety of cellular processes, including the cell cycle, apoptosis, cell proliferation, DNA damage response, autophagy, memory formation and migration and invasion [125,126]. BET proteins enhance the oncogenic functions of major cancer drivers by elevating the expression of these drivers, such as c-Myc in multiple myeloma, androgen receptor (AR) and ETS-related gene (ERG) in prostate cancer, and TWIST in breast cancer [127]. BRD4 is frequently overexpressed and clinically associated with a variety of human cancers. BRD4 is also under ubiquitination-mediated degradation. Cullin-3-SPOP earmarks BET proteins, including BRD2, BRD3 and BRD4, for ubiquitination-mediated degradation. SPOP is frequently mutated in primary prostate cancer. Prostate cancer-associated SPOP mutants fail to interact with and promote the degradation of BET proteins, leading to their increased abundance and causing a resistance to BET inhibitors in SPOP-mutant prostate cancer [125,128,129]. The E3 ligase substrate receptor cereblon (CRBN) promotes proteosomal destruction by engaging the DDB1-CUL4A-Roc1-RBX1 E3 ubiquitin ligase. Interestingly, dBET1, a chemical compound and targeting ligand, degrades BRD4 by stimulating CRBN’s E3 ubiquitin-conjugating function [130]. The degradation of BRD4 can be mitigated by the deubiquitinase DUB3 [131]. DUB3 binds to BRD4 and promotes its deubiquitination and stabilization. DUB3-proficient prostate cancer cells are resistant to the BET inhibitor JQ1 in vitro and in mice.

## 6. Conclusions and Perspectives

Aberrant profiles of histone modifications result in a variety of pathological diseases. The crosstalk between the ubiquitin and histone modifying enzymes and the biochemically reversible nature of histone modifications provides a platform for rapid changes in the activity of downstream targets. The dynamic changes of histone modifying enzymes by UPS form a sophisticated and regulated network to coordinate the plasticity and dynamic change required for cell homeostasis (Table 1).

Because of the rapid progress and appreciation for epigenetics, many factors that regulate the UPS for histone modifying enzymes have been identified. However, more questions have been raised than have been answered. First, what and how do extrinsic cellular signals trigger the degradation of histone modifying enzymes? Second, since most lysine residues function as acceptor sites for ubiquitination, acetylation and methylation, how do these different modifications impact the availability of histone modifying enzymes? Additionally, how do these histone modifying enzymes regulate E3 ligase through different histone modifications? Third, some of the histone modifying enzymes are degraded in the cytoplasm, whereas others are degraded in the nucleus, and the same enzyme can be degraded by different E3 ligases either in the cytoplasm or nucleus. Therefore, how do these E3 ligases cooperate to efficiently control concentrations of these enzymes to meet the requirements for individual cellular homeostasis? Fourth, although specific ubiquitin E3 ligases mediate the ubiquitination of a specific histone modifying enzyme, several histone modifying enzymes share the same E3 ligases. Can these modulators of the UPS be used clinically as therapeutic strategies by altering the abundance of these histone modifying enzymes? Finally, DUBs counteract E3 ligase activity and prevent ubiquitination-mediated degradation. How do these enzymes keep the balance to fine-tune the protein level of histone modifying enzymes?

In all, advances in our understanding of the crosstalk between histone modifying enzymes and the UPS can identify bi-stable switches that allow a dynamic regulation of gene expression states. This understanding will also provide a better sense of the molecular mechanisms associated with histone modification and transcriptional activity, and thus accelerate the development of new therapeutic strategies that target UPS to control PTMs in cellular homeostasis and disease.

## Figures and Tables

**Table 1:** The ubiquitin proteasomal regulation of histone modification enzymes.

Class of Histone Modifying Enzyme	UPS-Regulated Histone Modifying Enzyme	Target of the Histone Modifying Enzyme	Kinases Involved in UPS-Regulation of Histone-Modifying Enzyme	E3 ligases Regulating Histone Modifying Enzyme	DUBs Regulating Histone Modifying Enzyme	Physiological and Pathophysiological Functions of UPS-Regulation of Histone Modifying Enzyme
Acetylation Enzyme	p300	H2AK5, H2B, H3, H4		Mdm2, FBX3, BRMS1		Cell growth, proliferation, development, differentiation, cell-cycle regulation, DNA damage response, tumorigenesis and apoptosis
	PCAF	Free H3, H3K14, H4K8		Mdm2		proliferation, differentiation, apoptosis and cell-cycle progression
	HBO1	H3K14, H4	ATM/ATR	CRL4		cell-cycle progression, DNA replication and proliferation
			Mek1	FBXW15		
	Tip60	H2AK5, H4K16		UHRF1, EDD1, Mdm2	USP7	Cellular signaling, DNA damage repair and transcription
Deacetylation Enzymes	HDAC1	H2A, H2B, H3, H4		Mdm2, Chfr, REN		Development, gene repression, cell cycle, DNA repair, etc
	HDAC2	H2A, H2B, H3, H4K16		RLIM, Mule	USP4, USP17	Differentiation and Development, gene repression, cell cycle, DNA repair, etc
	SETD2	H3K36		SPOP		Transcription elongation, RNA processing, DNA repair and damage response, polycomb silencing
Lysine Methylation Enzymes	SETD3	H3K36	GSK3β	FBXW7β		Cell proliferation and tumorigenesis
	Set8	H3K20		Cdt2		Cell cycle progression, transcription regulation, DNA repair, genome stability and tumor metastasis
			CDK1	Cdh1		
				Skp2		
			CK1	β-TRCP		
	EZH2	H3K27		Smurf2, CHIP	USP21, ZRANB1	Cell proliferation, tumorigenesis, metastasis and stem cell renewal and maintenance
			Jak2	β-TRCP		
			CDK5	FBXW7		
			p38	Praja1		
Lysine Demethylation Enzymes	JMJD2A	H3K9, H3K36		FBXL4, FBXW2, FBXO22, RNF8		Replication timing and gemomic stability, DNA damage response, cellular differentiation, and animal development
	LSD1	H3K4		Jade2	USP28, USP22, USP7	Differentiation, self-renewal and tumor metastasis
Arginine Methylation Enzymes	PRMT1	H4R3		FBXL17, TRIM48, E4B, CHIP		Cell proliferation, progenitor maintenance and tumor metastasis
	PRMT4	H3R17, H3R26		Skp2		Transcription pre- mRNA splicing and cell cycle progression
Reader	BRD4			SPOP, CRBN	DUB3	Cell-cycle, apoptosis, cell proliferation, DNA damage response, autophagy, memory formation and migration and invision
